# Regulatory Considerations on the use of Machine Learning based tools in Clinical Trials

**DOI:** 10.1007/s12553-022-00708-0

**Published:** 2022-11-07

**Authors:** Maurizio Massella, Diego Alejandro Dri, Donatella Gramaglia

**Affiliations:** 1 Clinical Trials Office, Italian Medicines Agency (AIFA), Via del Tritone 181, 00187 Rome, Italy; 2 Department of Drug Chemistry and Technology, Sapienza University of Rome, Piazzale Aldo Moro 5, 00185 Rome, Italy

**Keywords:** Artificial intelligence, Big data, Clinical trials, Digital health, Machine learning, Regulatory

## Abstract

**Background:**

The widespread increasing use of machine learning (ML) based tools in clinical trials (CTs) impacts the activities of Regulatory Agencies (RAs) that evaluate the development of investigational medicinal products (IMPs) in clinical studies to be carried out through the use of data-driven technologies. The fast progress in this field poses the need to define new approaches and methods to support an agile and structured assessment process.

**Method:**

The assessment of key information, characteristics and challenges deriving from the application of ML tools in CTs and their link with the principles for a trustworthy artificial intelligence (AI) that directly affect the decision-making process is investigated.

**Results:**

Potential issues are identified during the assessment and areas of greater interaction combining key regulatory points and principles for a trustworthy AI are highlighted. The most impacted areas are those related to technical robustness and safety of the ML tool, in relation to data used and the level of evidence generated. Additional areas of attention emerged, like the ones related to data and algorithm transparency.

**Conclusion:**

We evaluate the applicability of a new method to further support the assessment of medicinal products developed using data-driven tools in a CT setting. This is a first step and new paradigms should be adopted to support policy makers and regulatory decisions, capitalizing on technology advancements, considering stakeholders’ feedback and still ensuring a regulatory framework on safety and efficacy.

**Graphical Abstract:**

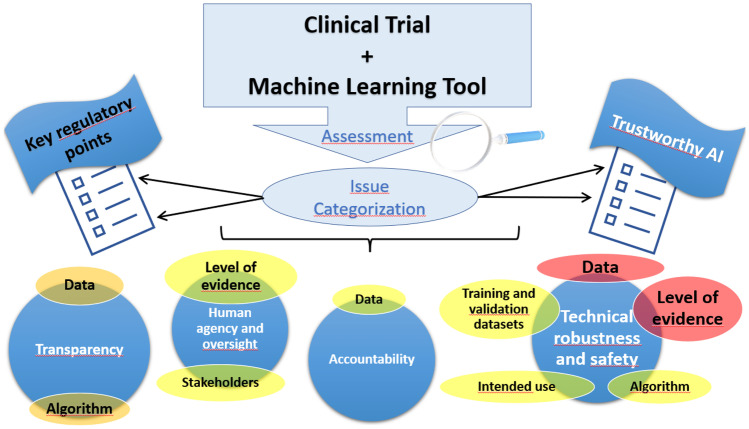

## Introduction

The availability of huge amount of data, together with an extraordinary computational power, is promoting a wide use of AI and at the same time it is raising ethical and social concerns that must be addressed to optimize the benefits and to prevent risks. The importance of the social role played by AI is described in the definition released by the Organisation for Economic Co-operation and Development [1], that verbatim quote: *“AI system is a machine-based system that can, for a given set of human-defined objectives, make predictions, recommendations, or decisions influencing real or virtual environments. AI systems are designed to operate with varying levels of autonomy”*. The use of AI and more in general of digital technologies is causing a global socio-economic change that also affects medicine and healthcare and this can be verified by the growing interest and by the number of scientific publications [2] of researchers and players attracted by AI and digitalization. By simply performing a Google Scholar review article search [3], we can retrieve more than 26,000 articles with a searching criteria “artificial intelligence healthcare” and more than 62,000 articles with a searching criteria “artificial intelligence medicine”. Health digitalization is a broad definition encompassing various technologies, ranging from apps for smartphones used to reveal skin cancer [4], or telemedicine often used during Covid-19 pandemic as measures to mitigate risks [5], to digital therapies [6] that are being developed and that impact on the behavior, like the first game-based digital therapeutic to improve attention function in children with attention deficit hyperactivity disorder (ADHD) approved in the United States (US). Digitalization can be found to be involved also in the clinical development processes of medicines where the use of ML, a discipline encompassed by AI and based on the use of mathematical modelling to analyze data, is providing important insights in term of prediction or classification by adapting the performance of the algorithm as the availability of data increases, obtaining important and significant gain of resources. The importance of digital technologies and AI is shown also by the increasing number of ML-based tools approved by the food & drug administration (FDA) [7] and by the fact that it is considered as a strategic goal in the european medicines agency (EMA) regulatory science to 2025 strategic reflection paper [8]. Regulatory Agencies (RAs) together with political institutions and scientific organizations are working and discussing about new paradigms, that are bringing along concerns, but with the aim to foster scientific research, and to accelerate the access of patients to therapeutic opportunities, still ensuring strong regulatory requirements. For instance, the international medical device regulators forum (IMDRF) [9] has released a framework for risk categorization based on the importance of the information provided and on the seriousness of the clinical condition, while FDA is giving various substantial contributions, for instance with the promotion of good machine learning practice (GMLP) [10], referred to data management and selection, training and tuning for building reliable software. The EMA and the heads of medicines agencies (HMA) have published two reports [11, 12] focused on the regulatory validity of big data, with the definition of various steps such as data standardization and evidence generation. The need to test the ability of ML methods to identify data that may support with the interpretation of healthcare data together with real-world data (RWD), in a clinical trial setting, is clearly identified by EMA in the recent regulatory science research needs publication [13]. The application of ML in CTs may widely vary, from patient recruitment to study design, to the definition of endpoints or to perform a more accurate diagnosis; in any case the assessment of these technologies is impacting the activities of RAs involved in the authorization of clinical studies [14﻿]. From a regulatory point of view, the lack of dedicated guidelines and harmonized approaches brings uncertainty among applicants and RAs [15], making it difficult to frame these tools, that sometimes according to the stated intended use, may be used within a trial for instance in the selection of patients to be enrolled, with the aim to just save resources in time consuming processes, and so they may not meet the definition of medical device. However, ML tool software should in principle be considered as a medical device, providing information which are even used by physicians for decision-making purposes with a therapeutic or diagnostic aim, and therefore e.g. in the EU, these are regulated under the Medical Device Regulation (EU) 2017/745 (MDR) [16] or the In Vitro Diagnostic Medical Devices Regulation (EU) 2017/746 (IVDR) [17]. Although the regulatory assessment of a medical device may be performed by a different office from the clinical trials one, or by a different RA in some Member States (MSs) in EU, the interaction between RAs, offices and assessors is mandatory. It is crucial to share data and information, and to ensure the compliance with fundamental principles such as the protection of rights, safety, dignity and well-being of subjects, and the generation of reliable and robust data in accordance to requirements set out in the Regulation (EU) 536/2014 [18] in EU or, in case of other countries not in the European Economic Area (EEA), in compliance with those principles described in the International Council of Harmonisation of Technical Requirements for Pharmaceuticals for Human Use (ICH) [19], Guideline for Good Clinical Practice [20] and the Declaration of Helsinki [21]. There is a clear need to standardize the regulatory approach to the assessment of ML tools in CTs to support a prompt regulatory acknowledgement and speed-up the incorporation of innovation into the CTs assessment and authorization process. We propose a step forward in such a process by discussing the requirements for a trustworthy AI and their relationship with the required key regulatory points and characteristics that need to be addressed for CTs that use ML-based technologies, focusing mainly on adaptive algorithms, as considered potentially more critical in a CT setting. However, the same approach and principles ma﻿y apply to the use of any ML/AI. We also analyze how issues identified during an assessment process could potentially impact both on crucial regulatory requirements and the requirements for a trustworthy AI, highlighting critical areas of interaction.

## Materials and methods

To identify the key points that need to be addressed in a CT setting involving the use of AI or ML systems, we took as a reference the first supporting guide available in the overall EU regulatory landscape specifically focusing on the request for authorisation and assessment of clinical trials involving the use of AI/ML [22]. Even if the guide may reflect one Competent Authority (CA) perspective, it is the only one that to the best of our knowledge lists and describes the regulatory information that should be submitted to the CA to request the authorization of a CT impacted by ML-based tools. The Ethics Guidelines for Trustworthy AI publication by the European Commission defines instead the key requirements for a trustworthy AI [23]. The standard assessment process of a CT is oriented in order to identify to what extent some key regulatory points are impacted, focusing on those primary areas of interaction with the requirements for a trustworthy AI as well as potential challenges that may be further elaborated and extrapolated to implement dedicated policies able to support the regulatory assessment and decision-making process in a real CT setting.

### ML predictive model

**Fig. 1:**
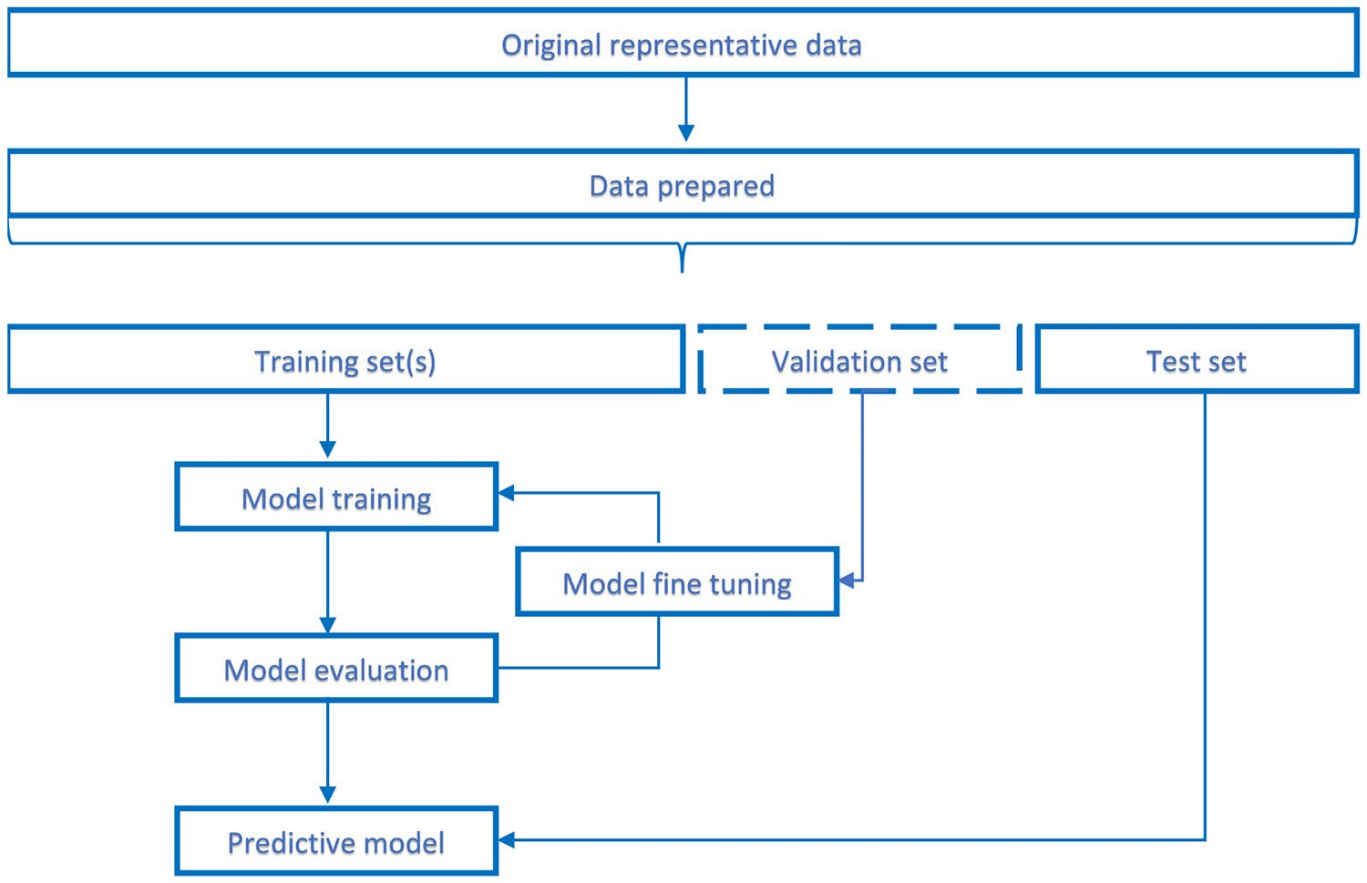
The ML predictive model development process

The output generation by a ML-tool derives from a complex process that could be basically and schematically described by the critical steps of the development process of the algorithms, such as training and validation. With the training process, the relationship between input and output parameters is coded and starting from the input, by an inferential mechanism, the model generates an output; this value is then compared with the true value, and the difference guide the update of the model. In this manner over time, the model learns to recognize the input, and it is possible to obtain the desired output with an acceptable precision. The training model depends on data and on its quality, including the representativeness, crucial to allow any machine learning algorithm to learn. As shown in Fig. 1, three data sets are usually needed, the training set, the validation set, and the testing set, allowing the training, the fine-tuning of the model, and the testing. Prognostic variables of the same specific patient population enrolled in the CT would be the original representative dataset that need to be prepared and fit for purpose to support the training and validation process of the ML predictive model.

### Key points to address in a CT setting involving the use of AI or ML systems

#### Data (DTA)

a data management plan including the type, the origin and method of acquisition of the data used, the reliability, security, standardization of the dataset(s), potential biases, and how potential low-quality data are intended to be managed.

#### Algorithm (ALG)

the type of result expected by the use of the software should be reported together with the version of the algorithm and a comparison with previous experiences and available tools.

#### Output (OTP)

the definition of what the machine generates together with the correlation to the scope, the objectives and/or endpoints of the CT. If it is a decision support software, an explanation of how the algorithm works in making decisions is expected, unless a proper level of access is provided to the CA.

#### Health care and clinical setting (HCS)

availability of statements of the clinical and epidemiological characteristics of the pathological condition, taking into considerations potential subgroups of patients, together with the description of standards of care routinely employed into clinical practice.

#### Intended use (INU)

the purpose and the intended use of the tool according to the statement of the manufacturer on the label (CE mark), if available, or in the protocol, including the added value (benefits) for patients in the context of the specific CT. Should the tool be a decision support software this condition should clearly be considered in a specific risk assessment, and it should be demonstrated and confirmed that the tool is safe and appropriate for the intended use.

#### Stakeholders (STK)

who are the users of the ML tool in the CT setting (healthcare personnel, subjects in the CT, etc.) and the compliance with the general data protection regulation (GDPR).

#### Level of evidence (LOE)

the intrinsic strength of the results of clinical studies deriving from scientific research used to build the model, and of the CT study results.

#### Training and validation datasets (TVD)

details on the representativeness of the training and validation datasets, provided together with information on the suitability of data, that is the capacity to answer to the clinical question taking into consideration potential bias and data collection methods [24]; proof of the independence of the training and of the test sets.

#### Performance metrics (PFM)

data on the performance of the model such as the area under the curve (AUC) and on the impact into the clinical setting, such as sensitivity, specificity, positive or negative predictivity, along with all statistic plans.

#### External validation / reproducibility (EVR)

the possibility of the model’s results to be generalized and reproduced, that also means the ability to obtain the same results by an independent assessor. Any possibility or additional method available to access the datasets and the model (by the CA, the subjects in the CT, the public, etc.).

#### Technologies and infrastructures (TAI)

data storage and cybersecurity, usability, data governance, hardware/interface requirements, data transmission, net connection etc.

**Fig. 2:**
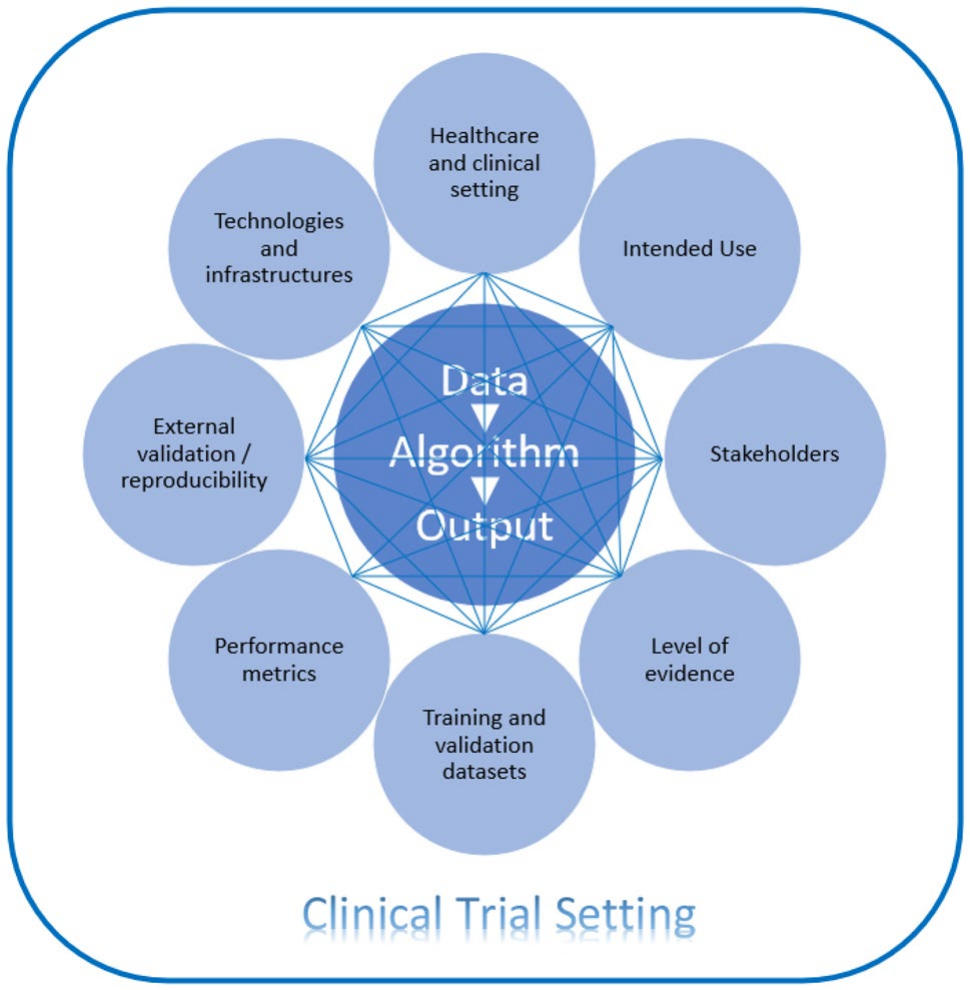
Data, algorithm, output and the relationship across key points impacted in a CT setting

In Fig. 2 it is illustrated the relationship among the core data, algorithm, and output, with the key points impacted in a clinical trial setting.

### Requirements for trustworthy AI in CTs

#### Human agency and oversight (HAO)

Respect of autonomy and of decisional process of human beings that should be ensured by the human oversight with measures through governance mechanisms.

#### Technical robustness and safety (TRS)

Development of system with a preventative approach to risks or threats of cyberattacks, minimizing unintentional and unexpected harm where possible.

#### Privacy and data governance (PDG)

Procedures for quality and integrity of data used and to process data in a manner that protects privacy.

#### Transparency (TRN)

Description of traceability mechanisms and the capacity to be fully understood, in term of functionality and operations by a person without any skill in AI.

#### Diversity, non-discrimination and fairness (DNF)

Development of system without discriminatory biases against certain group of patients.

#### Environmental and societal well-being (ESW)

AI should be used to benefit all human beings, including future generations.

#### Accountability (ACC)

Development of system able to ensure responsibility of various players throughout the AI system’s life cycle.

### Assessment

According to the Regulation (EU) 536/2014 [18] in EU, the Draft Assessment Report (DAR) of Part I, that include the assessment of the scientific documentation of the CT application dossier, consists of seven parts: introduction, quality assessment, pre-clinical assessment, clinical assessment, statistical methodological assessment, regulatory assessment, conclusion [25].

CT applications are always evaluated by a multidisciplinary team of assessors, whose composition is reported in Table 1.

**Table 1:** Multidisciplinary team involved in the assessment of a CT

**Multidisciplinary team**	
Assessor	Regulatory (including legal advisory if needed)
	Pre-clinical
	Clinical
	Quality
	Statistical methodological (including data scientist if needed)
Management	CT office manager

The regulatory assessor is responsible for ensuring the compliance with applicable laws and regulations of the CT application submitted by sponsors, should legal issues be identified, legal advisory is also included; the pre-clinical assessor assess the pharmacological properties such as pharmacodynamics and pharmacokinetics, comparative physiology and the toxicological profile of a drug in development stage; the clinical assessor is a physician that mainly focus the assessment activity on the CT protocol and related procedures, the clinical setting, the endpoints of the study, the population characteristics and the therapeutic area involved; the quality assessor may be a chemist, pharmacist, biologist depending on the characteristics of the investigational medicinal product (IMP) and is assessing the chemistry, manufacturing, and control (CMC) information provided by the sponsors to support the quality profile of the tested drug; the statistical assessor is focusing the assessment on the statistical analysis plan of the protocol and, when AI/ML is involved, data scientist competencies are required. If ﻿a final positive conclusion on all the parts of the DAR is achieved and an overall positive benefit–risk profile for the CT can be ensured, a final positive decision on the application can be taken. A multidisciplinary team of five assessors currently working at the clinical trials office (CTO) of a CA, that already assessed CTs impacted by the use of AI/ML, was involved in the assessment of the case in study.

We have considered in the assessment, the key points to address in a CT involving the use of AI or ML systems and the requirements for a trustworthy AI. Each of the five assessors were asked to provide, capitalizing on their experience and expertise, their input in terms of potential issues that may arise during the assessment of a CT that involves the use of AI/ML. They were also asked to focus only on those criticalities related to the key points and the requirements for a trustworthy AI. The outcome of the assessment is a list of potential issues. Any potential issue identified is then linked both to the key point impacted and to the requirements for trustworthy AI in CTs, using the issue categorization form available in the ﻿Appendix (Table 4). The association with the impacted key points and the requirements for a trustworthy AI is carried out independently and autonomously by the assessors, according to their best knowledge and belief and, although this may be considered a subjective evaluation, it has to be considered unquestionable for the purpose of this exercise, as it is the output of a scientific evaluation of an expert in the field. Reference to the assessment list for trustworthy AI (ALTAI) methodology [26] contributed to the description of the impact and challenges that a ML tool applied to a CT.

## Results

A list of 33 potential issues was identified, reported in Table 2.

**Table 2:** List of potential issues identified

**Potential issues**	**Key point associated**	**Requirements for trustworthy AI associated**
Lack of communication and explainability to patients and physicians. Otherwise use of software.	INU	TRN
Lack of human oversight. Prescription of sub-optimal SoC.	STK	HAO
Selection of patients. Non-responders.	PFM	TRS
Lack of external validity. Limitation in applying the results outside of the trial design and population.	LOE	TRS
Biased estimators of safety and efficacy of IMP.	TVD	TRS
Exclusion of patient that potentially could benefit from the IMP tested in the trial.	DTA	DNF
Misunderstanding of output results by physicians and patients.	DTA, TAI	TRN
Failure to respect fundamental rights.	STK	PDG
Poor quality of clinical trial data. Data accuracy.	DTA	PDG
Lack of access to trial data by the health Competent Authority.	DTA	ACC
Threats and inappropriate use of clinical trial data.	TAI	TRS
No details provided on the development process of the ML tool. The tool has not been fully validated.	TVD	TRS
No or limited information provided on the databases. The reliability of the datasets cannot be guaranteed.	DTA	TRS, TRN
No CE mark provided. The quality and performance of the algorithm is not ensured.	ALG, INU	TRS
No description of how the algorithms generates the output. The transparency of the clinical evidence generated cannot be ensured.	ALG, OTP, LOE	HAO, TRN
The Competent Authority is not given access to the development phase data and to the algorithms source. The quality of the clinical evidence generated cannot be ensured.	DTA, ALG, LOE, TVD, EVR	HAO, TRS, TRN
Predictive model autonomously taking decisions without the investigator oversight. SoC chosen based on the biomarkers may not be the one the Investigator may choose considering other patient conditions and information.	OTP, STK	HAO, DNF, ACC
Investigator may not be able to react on missing data. Data accuracy.	DTA, STK, LOE, EVR	HAO, TRS
Biomarker data are collected out of a controlled and standardized environment such as a clinical trial. Data accuracy.	DTA, HCS, LOE, TVD	HAO, TRS
Subject data can be disclosed. Data breach.	OTP, INU, TAI	TRS, PDG
Not the same population used in datasets for validation and training, and for the inclusion in the clinical trial. Data representativeness.	DTA, OTP, HCS, LOE	DNF
Not capitalizing on results of a clinical trial by disseminating the results openly and transparently in a timely manner. Missing information.	DTA, OTP, INU, EVR	TRN, DNF, ESW
Training and access control not ensured throughout all the clinical trial and AI development cycle. Mishandling of data and technology.	INU, TAI	TRS, ACC
Prognostic biomarkers not validated. Validity of scientific results.	DTA, LOE	TRS, ACC
Databases for biomarkers collection not standardized. Data accuracy.	DTA, LOE	TRS
Patient identified as good responder for both SoC1 and SoC2. Bias in statistical elaboration.	LOE, EVR	TRS, DNF
Benchmark and ranges to identify a good responder not provided. Scientific validity and ethical.	ALG, LOE	TRS, TRN
Added value in the use of the tool not clear or not described. Scientific validity and ethical.	INU, LOE	DNF, ESW, ACC
Versioning of the software not provided. No versioning control.	ALG	TRN, ACC
No clear identification of the users of the tool. Missing training or accountability.	STK	PDG, ACC
Data management plan not provided. Data accuracy.	DTA	TRS, TRN, ACC
Risk assessment regarding the use of the tool as a decision support software not available. Patient safety.	ALG, INU, STK	HAO, TRS, TRN, ACC
Databases used to store data not specified. Potential data breach and security.	DTA, TAI	PDG, ACC

Potential issues were linked to the requirement for trustworthy AI and impacted key points. For a given potential issue identified during the assessment, one or multiple key points as well as one or multiple requirements for trustworthy AI were associated using the issue categorization form (Table 4). The absolute number of issues impacting each single area after linking them both to the key point impacted and to the requirements for trustworthy AI in CTs, is reported in Table 3.

**Table 3:** Number of issues per key point and per requirements for trustworthy AI identified

**Key point impacted**	**Number of issues identified**	**Requirements for trustworthy AI impacted**	**Number of issues identified**
Data	14	Technical robustness and safety	18
Level of evidence	11	Accountability	10
Intended use	7	Transparency	10
Technologies and infrastructures	5	Human agency and oversight	7
Algorithm	6	Diversity, non-discrimination and fairness	6
Output	5	Privacy and data governance	5
Stakeholders	6	Environmental and societal well-being	2
External validation / reproducibility	4		
Training and validation datasets	4		
Healthcare and clinical setting	2		
Performance metrics	1		

**Fig. 3:**
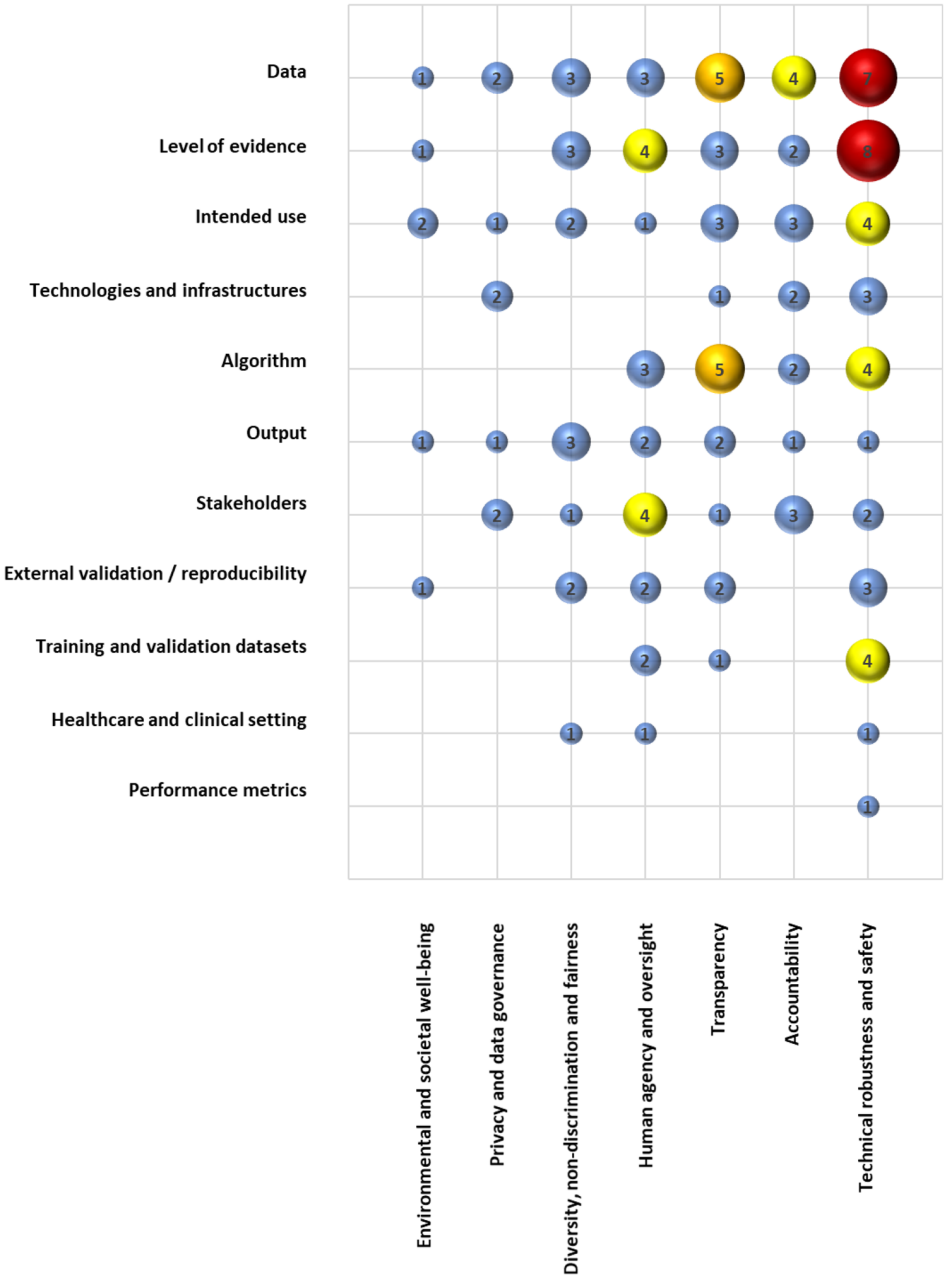
Number of issues impacting combined key points and requirements for trustworthy AI, highlighting the greater issue combination areas

The highest number of potential issues were identified in the fields of technical robustness and safety of the ML tool and in relation to the data. However also the level of evidence, data accountability and transparency are greatly impacted. Results were further elaborated to map them in terms of interactions between the key points and the requirements for trustworthy AI, highlighting the most impacted issue combination areas as reported in Fig. 3.

## Discussion

The use of ML-based tools or AI is an opportunity as far as it is trustworthy. Fulfilling this requirement in the context of a CT setting means to be able to provide a contribution to the safety and efficacy profile of the study, whose evaluation is the main task of RAs [27]. For this reason, the assessor at the CTO should know the tools employed into the clinical study, regardless of whether it meets the regulatory definition of medical device or not. In the assessment process, the key information submitted to RAs should be evaluated taking into consideration risks and benefits and how they may affect the main requirements for trustworthy AI. The findings resulting from the application of the assessment process, collected in the issue categorization form, contain some limitations like the potential lack of a strong evidence because of the methodological bias. Our considerations are extrapolated from the experience in the assessment process of assessors and, we could not report detailed data of a specific study, providing their interpretation and explaining the reasons of a regulatory decision. It is also important to consider that the assessment of the CT is performed by one team of professionals only however, even if this is the regular process, it could be also useful to share and compare our insights with an enlarged group of assessors facilitating the identification of inferential correlations. Sharing similar projects with other RAs would be a desirable aim to reach harmonization in the assessment. Another limitation is the awareness that the issues reported in Table 2 cannot be considered as an exhaustive list, since additional information may emerge during the assessment of a real protocol, and because multiple types of CTs (complex trials, different therapeutic areas, different patient populations etc.) and designs could be approached. Although these limits, the results in Fig. 3 can provide significant suggestions on how to complement the assessment process currently followed for any CT application at the CTO and could support the output of the actual regulatory authorisation process by helping to focus on those most impacted issue combination areas, highlighting those spheres of potential greatest risk. In addition, this is valuable information that could be used to further elaborate the intrinsic value of the results retrieved, as detailed in the following subsections, in light of an extrapolation exercise to cover other study designs and ML tools. This method could be even used to support the drafting of a dedicated guideline on the assessment of CTs impacted by ML or AI tools.

The most impacted areas in terms of interaction, combining key regulatory points and the principles for a trustworthy AI, after the identification of potential issues during the assessment, are those related to the technical robustness and safety of the ML tool when connected to the level of evidence generated and the data used. However also the transparency of the algorithm and of the data are additional areas of greatest impact. There are also other combined areas highlighting how training and validation datasets, the algorithm and the intended use can directly impact on the technical robustness and safety of the tool. Stakeholders and the level of evidence are connected to human agency and oversight, and data management is impacting accountability. Important information is also that the performance metrics of the tool and the healthcare and clinical setting do not seem to be very critical information, at least in terms of quantitative number of potential issues. However, the independence from a clinical setting could be considered as a point in favor of the potential extrapolability of the used method. Even if there are also additional areas of interaction that may have a minor impact, a qualitative analysis of each issue should always be completed, and all issues should be further explored during the assessment process.

### Technical robustness and safety

The most impacted issue combination areas highlight the main role that is played by data used to inform the adaptive algorithms. In the assessment process, the central role of data is directly related to the technical robustness and safety. The accuracy is the capacity of the software to make a correct prediction, the inaccuracy of the output pose unintended risks, like the one to prescribe a suboptimal therapy with less clinical efficacy and more undesirable effects. The level of evidence generated by clinical studies used to support the development phase of the tool, and that also impact on the technical robustness and safety of the tool, ultimately depend on the quality of the data used that impact the final CT study results. Risks and accuracy are estimated by performance metrics, used to quantify the predictive capability of the ML-model. The choice of metrics should be strongly related to the endpoints and should be able to ensure safety, efficacy and then equity by an early and clear statement of pre-set thresholds to be met in order to satisfy the acceptability of the system and to prevent unacceptable risks giving a substantial contribution to the achievement of technical robustness that is one of the key requirements for a trustworthy AI. To mitigate these risks, it is desirable to schedule in advance a monitoring plan of the accuracy, over time, with the aim to verify that the outputs remain acceptable. The level of accuracy, expected when the output affects humans, like in the case of a clinical trial, is also depending on the agreement around the uniqueness of clinical data definition by investigators, so far, a univocal data can be interpreted in only one way, but this condition may not always be fulfilled. This is evident and relevant for robust outcomes such as overall survival, but in some clinical settings, endpoints may not be available, and so these should be established taking into consideration potential harms [28]. The variability among investigators should be minimized and it is desirable the use of open shared data as much as possible by the most important and experienced physicians skilled in a specific disease; in addition to the accuracy of the output, another potential bias could come from the clinical site selected for the trial, because if the software is built capitalizing on data from an highly specialistic hospital, the management of the output, that is, the recommended therapy of the hypothetical study, could be more difficult in clinical settings less experienced and with a lower level of specialization. Robustness is given also by the level of evidence [29] of the data used to develop the software and the predictive model. It means that preferably standardized and secure data from clinical studies methodologically valid should be used, providing results with a relative strength ranked as high and so able to prevent risk of limited generalizability. About safety the main issue is the risk of cyber-attack that potentially impact any AI machine that should be resilient in case of threats and a certification showing the compliance with specific security standards should be provided, with a clear statement of the timeframe expected to ensure security.

### Diversity, non-discrimination and fairness

Another potential bias could derive from representativeness of datasets that could contain disparities and when these are used to train the algorithm, it could lead to an over or under estimation of the results [30]﻿ and so to biased programs with reduced predictive accuracy that generate or exacerbate discrimination in subgroups of population, compromising the requirements of fairness. With regards to datasets, a clear distinction between clinical study data used as datasets for training purposes, and those used as validation datasets should be guaranteed, without data sharing and with an adequate representativeness that should be estimated by statistical tests describing the minimum level of acceptability among datasets.

### Transparency, human agency and oversight, accountability

Regarding datasets, it is interesting to note that from a meeting organized by FDA [31] the importance of the explainability emerged and so of the transparency for patients that should have information about the representativeness of data used to train algorithms and even their possible changes, particularly about the intended use. The requirements of transparency of data and trustworthy AI could support to increase the quality of the data pool limiting some bias, like the selection bias related to representativity of the sample, that consequently will influence also discrimination, and by extension, fairness discussed in the previous section. There is an important link between bias and discrimination, intrinsic to processes such as those of data gathering, data cleaning and data processing [32]. Furthermore, transparency can contribute to improve the scientific rigor of clinical trials that could be affected by a critical concern know as publication bias, when only positive results of clinical studies are published, generating data that could in turn potentially be used to train algorithms. Technologies based on algorithms have an intrinsic opacity that is reducing the ability to explain the technical processes and to fully understand the reasons driving the generation of complex outputs, also by scientists that have created the algorithm [33]; this characteristic, referred as black box [34], cause a lack of predictability and raise the fear of potential loss of human oversight and consequently a reduction of trustworthy in AI. The improvement of knowledge is fundamental for the human oversight over the machine that don’t have to undermine human autonomy, that means that it should be ensured human discretion and so the users have to be able to take an autonomous decision; the oversight mechanisms are different and are dependent on the clinical trial setting; however, a variety of methods is available, and many ones can provide insights on the decision-making process. In any case, the awareness among health professionals using AI system, that the output is the result of an algorithm decision is desirable, to make a more informed decision. This would mean that the investigators should be always able not to accept the output of the machine and to prescribe, if considered necessary, any other pharmacological treatment considered as optimal for the patient. The definition of the level of autonomy in the assessment process should be considered as crucial (in particular with regard to the intended use), and so different considerations should be done if the software is classified as not autonomous or fully automated [35] because of the different risks associated, that will define a different liability in case of medical error [36] and in general different legal concerns. Legal issues that can vary significantly by jurisdiction, and cybersecurity are in any case, not specifically taken into consideration in the present manuscript where we are mainly focusing on regulatory considerations.

Transparency is a task that should be reached also by highlighting corrections or rectification of erroneous data, all changes should be traceable [37] and so a procedure should be in place to keep an audit trail to verify and identify what and how data has been changed, also including any statistical transformation or handling. Traceability procedures, as well as accuracy of data, are consistent with the principles of GDPR stating that data can be corrected or rectified, and that nothing should be hidden, and that it should be in compliance with the relevant standards for data management and governance throughout the life cycle of the CT. To achieve full transparency, clear statements regarding some key points like output and intended use should be openly communicated to RAs.

The initial and univocal description allows to avoid risks of otherwise use of the software because of the self-learning process; moreover, acknowledging the purpose of the tool is fundamental to evaluate the correctness of the data used to reach the aim, avoiding the risks to collect additional data for different purposes like e.g. marketing, consistently with the GDPR principles, such as purpose limitation and data minimization. The purpose is important also to justify the appropriateness of the output in terms of clinical relevance and the time for storage of data that must be no longer than necessary to reach the scope, in accordance with the storage principle of GDPR. Availability of data and information on process management and on algorithms could be relevant as far as it may be kept confidential by companies; difficulties in allowing data sharing because of commercial confidential information and privacy protection are acknowledged however, the replicability of results as well as data access for an independent evaluation and for inspections by CAs can improve significantly the explainability of the software and specially the auditability in compliance with accountability requirements. In terms of regulatory requirements and confidential commercial information, a fundamental contribute could be provided by the availability of ML-based tools with adequate transparency, equity and fairness, and more in general with a disclosure mechanism, because it could increase the trust of physicians and patients towards new technologies and consequently could promote its optimal use. This issue needs to be addressed by all players, by the promotion of an early interaction with academia, researchers, enterprises with patient engagement and regulatory bodies because the lack of shared guidelines increases the discretion of single CAs, extolling the differences in interpretation processes and facilitating heterogeneous evaluation approaches and so restraining the translation of research into clinical practice and ultimately the protection of public health.

### Privacy and data governance

Trustworthy could be improved by implementing procedures to ensure the quality of data, having source documents providing evidence and substantiating the integrity of the data collected, as well as procedures to ensure the compliance with data protection regulations, consistently with requirement of privacy and data governance. Given the high complexity of the task of ML-powered tools, the optimal use is strongly dependent by technical skill of health professionals that have to manage the software and that should have an adequate experience to understand benefits and risks and in case of damages, must be able to implement an appropriate risks minimization plan. About digital skill of healthcare professionals, a commitment of public institutions and/or scientific societies is desirable, to promote courses for digital training and to increase the confidence of investigators with data-driven technologies.

### Environmental and societal well-being

The last requirement is the societal and environmental well-being, that in the case of CTs should consider the potential change in the relationship physician–patient, that is a fundamental interaction in the clinical practice that could affect patient’s physical and mental well-being. Any data or information aimed at supporting such a relationship could increase the trustworthy of AI.

## Conclusion

Digital health technologies are triggering a paradigm shift and consequently RAs should implement new methods and approaches to complement the assessment of the safety and efficacy profile of medicinal products developed using data-driven tools, where data used play a main role. Data can both inform the adaptive algorithms, able to optimize their performance over time, or can be used in locked algorithms that do not update themselves in presence of new data and generate always the same outputs. In any case, the advantages in optimizing the performance with the use of learning algorithms should be balanced with various potential biases whose evaluation is an emerging issue on which various health institutions and international organizations are currently working on. Although the important efforts and significant results obtained so far, when a ML-based tool﻿ is proposed in a CT setting, additional efforts should be made to achieve a global harmonization of the assessment process. Stemming from our assessment, we propose a concrete starting point providing regulatory considerations following a bottom-up approach, moving from the point of view of the assessors that already had on their desks and assessed CTs impacted by ML methods, to link the key regulatory information to general principles for a trustworthy AI. The regulatory authorisation of CTs that use ML-based tools is a challenging task for all RAs that need to identify new methods of assessment and paradigms. The initial contributes of this paper should be further explored by enlarging the pool of assessors and by extending the feedback collection to a multistakeholders’ platform including among others, additional RAs, Ethic Committees, sponsors of CTs and patients. Our insights show the interaction between key regulatory points impacted and the requirements for trustworthy AI, as designed by the potential issues identified during the assessment, highlighting the most impacted issue combination areas. There is a clear evidence of the importance of the data used, with its connected level of evidence, directly impacting the technical robustness and safety of the ML tool. The cruciality of the data and the algorithm transparency also highlights elements to take into considerations in the regulatory assessment process. Other areas of mutual involvement are those relating to the intended use, algorithm, training and validation datasets, technical robustness and safety. Further areas of interaction may have additional intrinsic value depending on the specific CT design and setting, therefore even if specific areas of attention are clearly indicated, none of the key regulatory points or requirements for trustworthy AI should be excluded during the assessment of a CT that foresee a ML-based tool.

## Data Availability

Data sharing is not applicable to this article as no additional data are available.

## Appendix

**Table 4:** Issue categorization form

**Key point** **impacted**		**Potential** **issue**	**Requirements for** **trustworthy AI in CTs**
☐ Data ☐ Algorithm ☐ Output ☐ Healthcare and clinical setting ☐ Intended use ☐ Stakeholders ☐ Level of evidence ☐ Training and validation datasets ☐ Performance metrics ☐ External validation / reproducibility ☐ Technologies and infrastructures		*Issue* *description*	☐ Human agency and oversight ☐ Technical robustness and safety ☐ Privacy and data governance ☐ Transparency ☐ Diversity, non-discrimination and fairness ☐ Environmental and societal well-being ☐ Accountability

## References

[1] Recommendation of the Council on Artificial Intelligence (OECD Legal Instruments. OECD/ LEGAL/O449. 2019. https://legalinstruments.oecd.org/en/instruments/OECD-LEGAL-0449. Accessed 09 April 2022.

[2] Morrison C (2019). AI developers tout revolution, drugmakers talk evolution. Nat Biotechnol.

[3] https://scholar.google.com/. Accessed 09 April 2022.

[4] Zakhem GA, Motosko C, Ho RS (2018). How Should Artificial Intelligence Screen for Skin Cancer and Deliver Diagnostic Predictions to Patients?. JAMA Dermatol.

[5] ISS. Gabbrielli F, Bertinato L, De Filippis G, Bonomini M, Cipolla M. Rapporti ISS COVID-19 n. 12/2020. 2020, ii, 29p. https://www.iss.it/web/guest/rapporti-covid-19. Accessed 09 April 2022.

[6] Waltz E (2020). First video game to treat disease gains FDA okay. Nat Biotechnol.

[7] Stan B, Pranavsingh D, Bertalan M (2020). The state of artificial intelligence-based FDA-approved medical devices and algorithms: an online database. NPJ Digital Medicine.

[8] EMA. European Medicine Agency. EMA regulatory science to 2025 https://www.ema.europa.eu/en/documents/regulatory-procedural-guideline/ema-regulatory-science-2025-strategic-reflection_en.pdf. Accessed 09 April 2022 .

[9] IMDRF. Software as a Medical Device (SaMD) Working Group. "Software as a Medical Device": Possible Framework for Risk Categorization and Corresponding Considerations. https://www.imdrf.org/sites/default/files/docs/imdrf/final/technical/imdrf-tech-140918-samd-framework-risk-categorization-141013.pdf. Accessed 09 April 2022.

[10] FDA. Food and drug administration. Artificial Intelligence and Machine Learning in Software as a Medical Device Action Plan. https://www.fda.gov/media/145022/download. Accessed 09 April 2022 .

[11] EMA. Europeam Medicines Agency. HMA-EMA Joint Big Data Taskforce. https://www.ema.europa.eu/en/documents/minutes/hma/ema-joint-task-force-big-data-summary-report_en.pdf. Accessed 09 April 2022.

[12] EMA. European Medicines Agency. ‘Evolving Data-Driven Regulation’. https://www.ema.europa.eu/en/documents/other/hma-ema-joint-big-data-taskforce-phase-ii-report-evolving-data-driven-regulation_en.pdf. Accessed 09 April 2022 .

[13] EMA. European Medicines Agency. Regulatory Science and Innovation. Regulatory Science Research Needs (version 1.0). 13 December 2021, EMA/705364/2021. https://www.ema.europa.eu/en/documents/other/regulatory-science-research-needs_en.pdf. Accessed 09 April 2022.

[14] Dri DA, Massella M, Gramaglia D, Marianecci C, Petraglia S (2021). Clinical Trials and Machine Learning: Regulatory Approach Review. Rev Recent Clin Trials.

[15] Minssen T, Gerke S, Aboy M, Price N, Cohen G. Regulatory responses to medical machine learning. Volume 7, Issue 1, January-June 2020, lsaa002. 10.1093/jlb/lsaa002. 10.1093/jlb/lsaa002 PMC8248979 34221415

[16] Regulation (EU) 2017/745 of the European Parliament and of the Council of 5 April 2017 on medical devices, amending Directive 2001/83/EC, Regulation (EC) No 178/2002 and Regulation (EC) No 1223/2009 and repealing Council Directives 90/385/EEC and 93/42/EEC. Official J Eur Union. https://eur-lex.europa.eu/legal-content/EN/TXT/?uri=CELEX%3A32017R0745. Accessed 09 April 2022.

[17] Regulation (EU) 2017/746 of the European Parliament and of the Council of 5 April 2017 on in vitro diagnostic medical devices and repealing Directive 98/79/EC and Commission Decision 2010/227/EU. Official J Eur Union. https://eur-lex.europa.eu/eli/reg/2017/746/oj. Accessed 09 April 2022.

[18] Regulation (EU) No 536/2014 of the European Parliament and of the Council of 16 April 2014 on clinical trials on medicinal products for human use, and repealing Directive 2001/20/EC. Official J Eur Union. https://ec.europa.eu/health/system/files/2016-11/reg_2014_536_en_0.pdf. Accessed 09 April 2022.

[19] ICH. International Council of Harmonisation of Technical Requirements for Pharmaceuticals for Human Use. https://www.ich.org/. Accessed 09 April 2022.

[20] EMA. European Medicine Agency. Guideline for good clinical practice E6(R2). EMA/CHMP/ICH/135/1995 Committee for Human Medicinal Products. https://www.ema.europa.eu/en/documents/scientific-guideline/ich-e-6-r2-guideline-good-clinical-practice-step-5_en.pdf. Accessed 09 April 2022.

[21] WMA. World medical association. Declaration of Helsinki. https://www.wma.net/what-we-do/medical-ethics/declaration-of-helsinki/. Accessed 09 April 2022.

[22] AIFA. Italian Medicines Agency. Guide to the submission of a request for authorisation of a Clinical Trial involving the use of Artificial Intelligence (AI) or Machine Learning (ML) systems. https://www.aifa.gov.it/documents/20142/871583/Guide_CT_AI_ML_v_1.0_date_24.05.2021_EN.pdf. Accessed 09 April 2022.

[23] EC. European Commission. High-Level Expert Group on Artificial Intelligence. https://ec.europa.eu/futurium/en/ai-alliance-consultation.1.html. Accessed 09 April 2022.

[24] Danish medicine agency. Suggested criteria for using AI/ML algorithms in GxP. https://laegemiddelstyrelsen.dk/en/licensing/supervision-and-inspection/inspection-of-authorised-pharmaceutical-companies/using-aiml-algorithms-in-gxp/. Accessed 09 April 2022

[25] EMA. European Medicine Agency. How to evaluate a Clinical Trial Application: Assessment and Decision. CTIS Training Programme – Module 08 Version 1.3 – May 2022. https://www.ema.europa.eu/en/documents/other/quick-guide-part-i-how-evaluate-initial-clinical-trial-application-assessment-decision-ctis-training_en.pdf. Accessed 07 October 2022.

[26] EC. European Commission. Assessment List for Trustworthy Artificial Intelligence (ALTAI) for self-assessment https://digital-strategy.ec.europa.eu/it/node/806. Accessed 09 April 2022.

[27] Rivera SC, Liu X, Chan AW, Denniston AK, Calvert MJ. The SPIRIT-AI and CONSORT-AI Working Group, SPIRIT-AI and CONSORT-AI Steering Group and SPIRIT-AI and CONSORT-AI Consensus Group. Guidelines for clinical trial protocols for interventions involving artificial intelligence: the SPIRIT-AI extension. Nat Med. 2020;1351–1363. 10.1016/S2589-7500(20)30219-3. 10.1038/s41591-020-1037-7 PMC7598944 32908284

[28] Abràmoff MD, Tobey D, Char DS (2020). Lessons Learned About Autonomous AI: Finding a Safe, Efficacious, and Ethical Path Through the Development Process. Am J Ophthalmol.

[29] Djulbegovic B, Guyatt GH. Progress in evidence-based medicine: a quarter century on. Lancet. 2017; 22;390(10092):415–423. 10.1016/S0140-6736(16)31592-6. 10.1016/S0140-6736(16)31592-6 28215660

[30] Char DS, Shah NH, Magnus D (2018). Implementing Machine Learning in Health Care—Addressing Ethical Challenges. N Engl J Med.

[31] FDA. Food and Drug Administration. Public workshop—evolving role of artificial intelligence in radiological imaging. 2020. https://www.fda.gov/medical-devices/workshops-conferences-medical-devices/publicworkshop-evolving-role-artificial-intelligence-radiological-imaging-02252020-02262020. Accessed 09 April 2022.

[32] Varona D, Suárez JL (2022). Discrimination, Bias, Fairness, and Trustworthy AI. Appl Sci.

[33] Rahwan I, Cebrian M, Obradovich N, Bongard J, Bonnefon JF, Breazeal C, Crandall JW, Christakis NA, Couzin ID, Jackson MO, Jennings NR, Kamar E, Kloumann IM, Larochelle H, Lazer D, McElreath R, Mislove A, Parkes DC, Pentland A, Roberts ME, Shariff A, Tenenbaum JB, Wellman M (2019). Machine behaviour. Nature.

[34] Chinzei K, Shimizu A, Mori K, Harada K, Takeda H, Hashizume M, Ishizuma M, Kato N, Kawamori K, Kyo S, Nagata K, Yamane T, Sakuma I, Ohe K, Mitsuishi M. Regulatory Science on AI-based Medical Devices and Systems. Adv Biomed Eng 2018;7:118–123. 10.14326/abe.7.118.

[35] Yang GZ, Cambias J, Cleary K, Daimler E, Drake J, Dupont PE, Hata N, Kazanzides P, Martel S, Patel RV, Santos, VJ Taylor RH. Medical robotics—Regulatory, ethical, and legal considerations for increasing levels of autonomy. Sci Robotics. 2017;2(4):eaam8638. 10.1126/scirobotics.aam8638. 10.1126/scirobotics.aam8638 33157870

[36] Bitterman DS, Aerts HJWL, Mak RH (2020). Approaching autonomy in medical artificial intelligence. Lancet Digit Health.

[37] EMA. European Medicines Agency. Guideline on computerised systems and electronic data in clinical trials. https://www.ema.europa.eu/en/documents/regulatory-procedural-guideline/draft-guideline-computerised-systems-electronic-data-clinical-trials_en.pdf. Accessed 09 April 2022.

